# Gut Microbial SNPs Induced by High-Fiber Diet Dominate Nutrition Metabolism and Environmental Adaption of *Faecalibacterium prausnitzii* in Obese Children

**DOI:** 10.3389/fmicb.2021.683714

**Published:** 2021-05-31

**Authors:** Hui Li, Liping Zhao, Menghui Zhang

**Affiliations:** ^1^State Key Laboratory of Microbial Metabolism and Joint International Research Laboratory of Metabolic and Developmental Sciences, School of Life Sciences and Biotechnology, Shanghai Jiao Tong University, Shanghai, China; ^2^Ministry of Education Key Laboratory for Systems Biomedicine, Shanghai Centre for Systems Biomedicine, Shanghai Jiao Tong University, Shanghai, China

**Keywords:** SNP, gut microbiota, high-fiber diet, non-synonymous, obese children, metagenome

## Abstract

Dietary intervention is effective in human health promotion through modulation of gut microbiota. Diet can cause single-nucleotide polymorphisms (SNPs) to occur in the gut microbiota, and some of these variations may lead to functional changes in human health. In this study, we performed a systematic SNP analysis based on metagenomic data collected from children with Prader–Willi syndrome (PWS, *n* = 17) and simple obese (SO) children (*n* = 19), who had better healthy conditions after receiving high-fiber diet intervention. We found that the intervention increased the SNP proportions of *Faecalibacterium*, *Bifidobacterium*, and *Clostridium* and decreased those of *Bacteroides* in all children. Besides, the PWS children had *Collinsella* increased and *Ruminococcus* decreased, whereas the SO had *Blautia* and *Escherichia* decreased. There were much more BiasSNPs in PWS than in SO (4,465 vs 303), and only 81 of them appeared in both groups, of which 78 were from *Faecalibacterium prausnitzii*, and 51 were nonsynonymous mutations. These nonsynonymous variations were mainly related to pathways of environmental adaptation and nutrition metabolism, particularly to carbohydrate and nucleotide metabolism. In addition, dominant strains carrying BiasSNPs in all children shifted from *F. prausnitzii* AF32-8AC and *F. prausnitzii* 942/30-2 to *F. prausnitzii* SSTS Bg7063 and *F. prausnitzii* JG BgPS064 after the dietary intervention. Furthermore, although the abundance of *Bifidobacterium* increased significantly by the intervention and became dominant strains responsible for nutrition metabolism, they had less BiasSNPs between the pre- and post-intervention group in comparison with *Faecalibacterium*. The finding of *F. prausnitzii* as important functional strains influenced by the intervention highlights the superiority of applying SNP analysis in studies of gut microbiota. This study provided evidence and support for the effect of dietary intervention on gut microbial SNPs, and gave some enlightenments for disease treatment.

## Introduction

Single-nucleotide polymorphism (SNP) is the most common genetic variation in DNA sequence in order to better adapt to the external environment in the evolutionary process (Haraksingh and Snyder, 2013). SNPs in the coding region can be classified as nonsynonymous mutation and synonymous mutation. Nonsynonymous mutation changes the sequence of amino acids and then affects the genetic function, while the synonymous mutation does not affect the genetic function (Tennessen, 2008). There are huge amounts of microorganisms living in the human intestinal tract, and the diet is one of the most important factors shaping the structure and function of the gut microbiota (Goldsmith and Sartor, 2014; Shen, 2017). Environmental pressure caused by the change in the diet not only alter the structure of the gut microbiota but also led to genetic variations in the microbes (Truong et al., 2017). These variations can lead to different functions in strains, which, in turn, affect the health of the host.

Considering the taxonomic diversity of bacteria and the genetic variations in response to constant environmental change (Hofreiter et al., 2015), the analyses based on the abundance and composition of the gut microbiota are not enough to reflect changes in gene function or microbial transfer, which might not only omit some correlations but also infer wrong conclusions from this rough quantitative level. For instance, researchers had studied the gut metagenomes of 98 mothers and their infants over 1 year, used rare SNPs to reveal vertical transmission of strains, and found that the colonization with strains of infants mainly derived from the environment but not from their mothers, although the gut microbial composition of infants converged toward that of their mothers over time (Nayfach et al., 2016). This pattern might be missed in the analysis with the gut microbial composition, and it was mistakenly assumed that vertical bacterial transmission of infants from their mother was increased during the first year after birth.

Single-nucleotide polymorphisms, which refer to single-nucleotide variations in genes, are more able to reveal the differences between strains and between genes. The continuous expansion of gut metagenomic sample dataset and an increasing number of the bacterial reference genome have facilitated the studies of gut microbial SNPs. The flexible application of gut microbial SNPs can solve the complex problems that other analysis at species level cannot solve, obtain more accurate results at the strain- or gene-level, and provide new clues for the precision diagnosis and treatment of diseases (Galloway-Peña et al., 2012; Leonard et al., 2016; Zou et al., 2020). Patient-specific SNPs were found in the gut microbiota of both type 2 diabetes mellitus (T2D) and tuberculosis patients, which could separate the patients from the healthy individuals. The gene carrying T2D-specific SNPs encodes the alpha glucoside enzyme, which is a kind of important T2D drug target, and the researchers believed that these SNPs could be used as drug targets for the treatment of T2D (Chen et al., 2017). The tuberculosis-specific SNP genes were mainly involved in carbohydrate metabolism prevalently from *Bacteroides vulgatus*, suggesting that there were altered carbohydrate preference and different carbohydrate metabolism patterns in the gut of tuberculosis patients, and providing reference for the diagnosis and treatment of tuberculosis (Hu et al., 2019). Other researchers conducted gut microbial SNP studies on antibiotic resistance genes of individuals from different countries and found that the population-specific SNPs on antibiotic resistance genes were not related to the country, but might be attributed to the altered microbiota by differences in population structure or different antibiotic usage (Hu et al., 2013).

Many studies have proved the close relationship between the gut microbiota and obesity, but in-depth researches on the strain- or gene-level still need to be conducted (Turnbaugh et al., 2006; Flint, 2011; Baothman et al., 2016). Our previous study demonstrated that a high-fiber dietary intervention significantly improved the physiological conditions of the genetic (Prader–Willi syndrome, PWS) and simple obese (SO) children, and this promotion was found to be relevant to the change in the gut microbiota (Zhang et al., 2015). In order to better understand the underlying mechanism for this effective treatment, we performed a systemic SNP analysis based on high-throughput metagenomic sequencing data obtained from two longitudinal cohorts, children with PWS or SO. We first identified SNPs in each cohort affected by the intervention and screened out genes with significant change in SNP density. The species that carried these genes were then sourced and linked with relevant metabolic pathways. After that, we focused on BiasSNPs that occurred in both cohorts, particularly on those nonsynonymous mutations. Functional pathways and dominant strains with BiasSNPs influenced by the intervention were investigated further. Finally, PWS and SO-specific BiasSNP-affected strains and metabolic pathways were individually analyzed.

## Materials and Methods

### Data Collection

The dietary intervention trial was approved by the Ethics Committee of the School of Life Sciences and Biotechnology, Shanghai Jiao Tong University, with No. 2012-016 and registered at the Chinese Clinical Trial Registry with No. ChiCTR-ONC-12002646. Written informed consent was obtained from the guardian of the obese children. The trial was performed as described in the previous study (Zhang et al., 2015). Briefly, 17 PWS and 19 SO children completed the dietary intervention in the hospital for 90 and 30 days, respectively. The diet used in the clinical trial for intervention mainly included whole grains, traditional Chinese medicinal foods, and prebiotics (WTP), incorporated certain amount of vegetables, fruits, and nuts (Xiao et al., 2014).

The fecal samples and physiological indexes of all obese children were collected at predefined time points (PWS: on intervention days 0, 30, 60, and 90; SO: days 0 and 30) (Zhang et al., 2015). Metagenomic sequencing of the extracted and purified DNA was performed on Illumina Hiseq 2000 platform at Shanghai Biotechnology Co., Ltd. All potential biologically hazardous materials in this study were properly handled according to Chinese biosafety laws and regulations. The raw metagenome sequencing data was accessed at the NCBI SRA (Sequence Read Archive) database with accession number SRP045211 (Zhang et al., 2015).

### Detection of Single-Nucleotide Polymorphisms

The data pre-processing was done as described in our previous study (Zhang et al., 2015). Briefly, the original sequencing data was quality controlled by FlexBar and Prinseq (Schmieder and Edwards, 2011; Dodt et al., 2012), and then aligned to the human genome reference (*Homo sapiens*, UCSC hg19) using Bowtie2 to remove the reads from human (Langmead and Salzberg, 2012). Each sample had 84.6 ± 21.2 million (mean ± SD) high-quality reads on average. Then BWA (Li and Durbin, 2009) was used to align the high-quality reads to the integrated gene catalog (IGC), which contained approximately 11 million high-quality human gut microbial reference genes (Li et al., 2014; Xie et al., 2016). Afterward, the SAMTools was used to detect, filter, sort, and merge SNPs (Li et al., 2009; Li, 2011).

To ensure the reliability of the detected SNPs, only SNPs with at least five supported sequencing reads were kept, and those with less than 60% coverage in a group were furtherly removed to achieve more representative SNPs. The downstream analyses were all performed with the SNPs resulting from this procedure.

### Calculation of Single-Nucleotide Polymorphisms Density

Single-nucleotide polymorphism density reflected the number of SNPs per kilobase in a gene per million sequencing paired-end reads. For any sample *S*, the SNP density *D_i_* of gene *i* was calculated as follows:

$${\text{D}}_{i}=\frac{{\text{n}}_{i}}{{\text{T}}_{S}\,{\text{L}}_{i}}$$

where *n_i_* is the number of SNPs in gene *i*, *T_s_* is the sequencing amount of sample *S*, and *L_i_* is the length of the gene *i* per kilobase in sequence.

### Detection of BiasSNP

Detection of BiasSNP was performed through comparison of an SNP in a nucleotide base between two groups. A BiasSNP was the differential SNP that dominated between two groups (Supplementary Figure 1). Nonsynonymous BiasSNP, whose mutation in a gene caused the encoded amino acid to be changed, was furtherly identified by in-house Perl script.

### Statistical Analysis

In this study, the significance of the difference was judged with Wilcoxon paired test for a cohort between before and after the dietary intervention or with Wilcoxon unpaired test for two cohorts in R software (version 3.5.3). The significance of the difference among multiple groups was tested with permutational multivariate analysis of variance (perMANOVA) in the “vegan” library of the R software. The phylogenetic and clustering trees based on BiasSNPs were constructed using the maximum likelihood model GTRGAMMA and 1,000 bootstrap replicates in RAxML (Stamatakis, 2014). Additionally, the R package “clusterProfiler” (version 3.8.1) was used to perform enrichment analysis of the SNP genes (Yu et al., 2012).

### Data Visualization

Data visualization was mainly realized in the R software, “ggalluvial” (version 0.11.3), which was used to illustrate the alluvial diagram between strain and metabolic pathway, while dotplot, boxplot, and pieplot were displayed by means of “ggplot2” (version 3.2.0). In addition, the network charts were drawn using the Cytoscape software (version 3.7.2) (Shannon et al., 2003), and the optimization of the tree diagram was accomplished with the aid of the online tool EvolView^1^.

## Results

### The Overall Effect of High-Fiber Dietary Intervention on Gut Microbial Single-Nucleotide Polymorphisms in Obese Children

**FIGURE 1:**
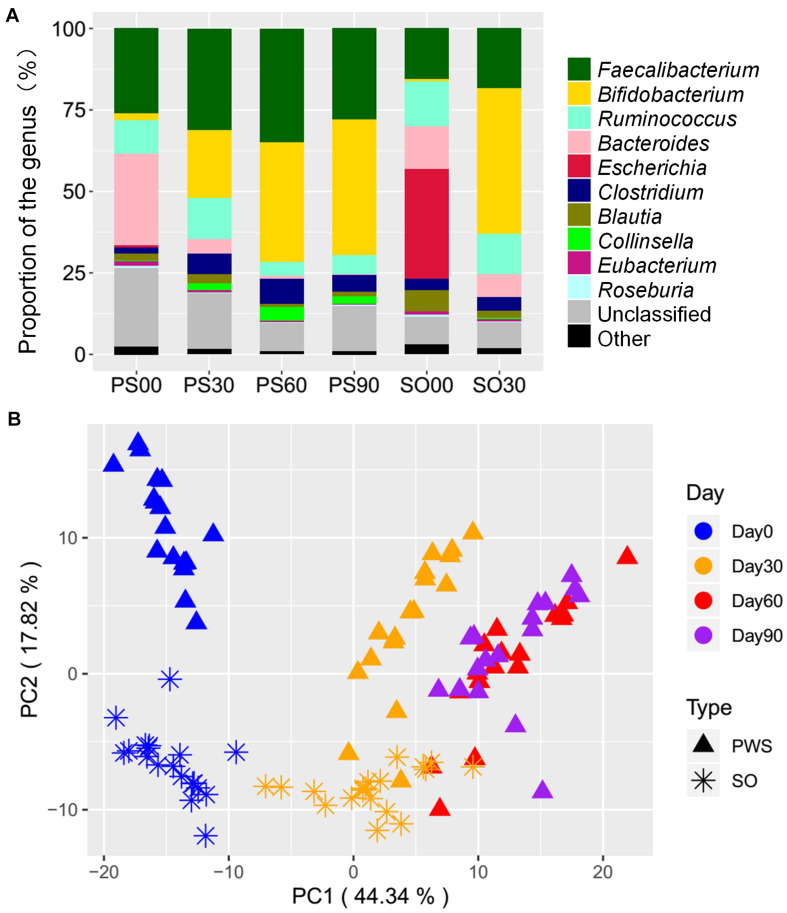
High-fiber dietary intervention altered gut microbial single-nucleotide polymorphism (SNP) pattern in both Prader–Willi syndrome (PWS) (*n* = 17) and simple obese (SO) (*n* = 19) children. **(A)** The composition of SNPs at the genus level among different interventional time points. PWS on day 0 (PS00), 30 (PS30), 60 (PS60), and 90 (PS90); SO on day 0 (SO00) and day 30 (SO30). **(B)** PCA plot based on the SNP density of gut microbiota.

A total of 218,343 SNPs were detected in the gut microbiota of the 36 obese children. These SNPs were concentrated in 40,515 genes, and these genes could be sourced from 57 genera and 150 species. The high-fiber dietary intervention lessened the overall SNP numbers on the 30th intervention day; the SNPs in PWS children decreased to 85,769 from 109,139, while in SO children, they decreased to 36,247 from 80,773. Before the intervention, all children had the dominant SNPs at genus level, which were from *Faecalibacterium*, *Ruminococcus*, and *Bacteroides*. In addition, the SO children had SNPs from *Escherichia* with relatively higher proportion (33.76%). The intervention increased the SNP proportions of *Faecalibacterium*, *Bifidobacterium*, and *Clostridium* and decreased those of *Bacteroides* in all children. Meanwhile, the PWS children had *Collinsella* increased and *Ruminococcus* decreased, whereas the SO had *Blautia* and *Escherichia* decreased (Figure 1A).

### The Altered Single-Nucleotide Polymorphism Density Structure in Prader–Willi Syndrome and Simple Obese Children

In our previous study, the PWS group had worse health conditions such as higher inflammation level than the SO group before the intervention. However, we did not detect significant difference between the two groups in gut microbial structure (Zhang et al., 2015). Interestingly, with the SNP density structure, a significant separation between the two groups before the intervention was observed (Figure 1B, PerMANOVA test, *P* < 0.001). The SNP density structure was altered significantly by the dietary intervention in both groups (Figure 1B, PerMANOVA test, *P* < 0.001). According to the changes that occurred in the PWS group, the structure alteration might occur mainly in the earlier stage of the intervention, as the shift on the 60th and 90th days was less that on the 30th day.

**FIGURE 2:**
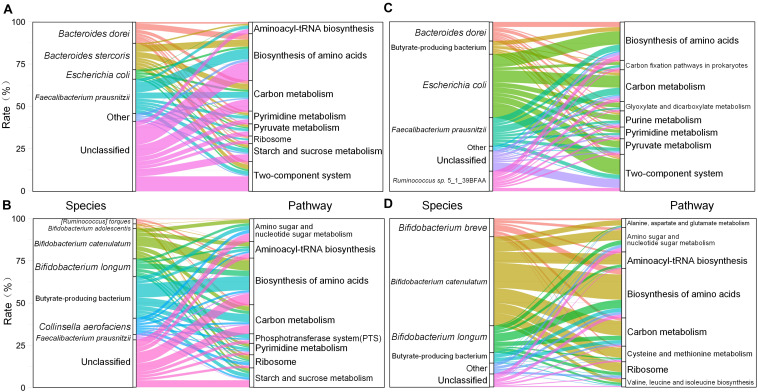
Species and metabolic pathways corresponding to the genes with differential SNP density between before and after intervention in obese children. **(A)** The corresponding species and metabolic pathways of the genes with high SNP density in PWS before the intervention. **(B)** The corresponding species and metabolic pathways of the genes with high SNP density in PWS after the intervention. **(C)** The corresponding species and metabolic pathways of the genes with high SNP density in SO before the intervention. **(D)** The corresponding species and metabolic pathways of the genes with high SNP density in SO after the intervention.

After 30 days of dietary intervention, the PWS children had 26,174 genes significantly changed in SNP density (Wilcoxon test, adjusted *P* < 0.05), and most of them (20,279) had fold changes lager than 8. Among these genes, 13,450 had higher SNP density before the intervention, which were distributed in 102 species and mainly concentrated in *Faecalibacterium prausnitzii* (20.27%), *Bacteroides stercoris* (15.63%), and *Bacteroides dorei* (12.49%). These genes were enriched in metabolic pathways for amino acid biosynthetic (ko01230), carbon metabolism (ko01200), two-component system (ko02020), and starch and sucrose metabolism (ko00500) (Figure 2A). After the intervention, 6,847 genes had higher SNP density. These genes were concentrated in 59 strains, mainly including butyrate-producing bacterium (24.65%), *Bifidobacterium catenulatum* (17.95%), and *Bifidobacterium longum* (10.60%). The corresponding metabolic pathways contained biosynthesis of amino acids (ko01230), carbon metabolism (ko01200), and starch and sucrose metabolism (ko00500) (Figure 2B). Notably, although the enriched metabolic pathways remain constant, their contributing strains changed after the intervention. For instance, the biosynthesis of amino acids (ko01230) was mainly from *F. prausnitzii* before the intervention, while the contributing strains of this function were replaced by butyrate-producing bacterium, *B. catenulatum*, and *B. longum* after the intervention.

The SO children had 17,427 genes significantly changed in SNP density (adjusted *P* < 0.05). The number of genes with fold change greater than 8 was 13,927. Among them, 10,409 genes with higher SNP density existed in the pre-intervention group, which were derived from 112 strains and mainly in *Escherichia coli* (37.53%), *F. prausnitzii* (16.88%), and *Ruminococcus* sp. 5_1_39BFAA (11.84%). Though the distribution of these strains in SO were different from that in PWS, these SNP density differential genes they carried also focused on biosynthesis of amino acids (ko01230), carbon metabolism (ko01200), and two-component system (ko02020) (Figure 2C), while 3,518 genes with higher SNP density were detected in the post-intervention group, which were derived from 26 strains and mainly in *B. catenulatum* (52.84%), *B. longum* (15.98%), and *Bifidobacterium breve* (10.83%). These genes focused on biosynthesis of amino acids (ko01230), carbon metabolism (ko01200), and amino sugar and nucleoside sugar metabolism (ko00520) (Figure 2D). Similar to the PWS, the intervention also changed the relationships between the strains and the metabolic pathways. However, unlike in PWS, *E. coli* followed by *F. prausnitzii* were the main contributors in SO to the biosynthesis of amino acids before the intervention, while contributions from these two strains might be neglected, and *B. catenulatum* took dominant responsibilities after the dietary intervention.

### Common BiasSNPs Before and After Intervention in Prader–Willi Syndrome and Simple Obese Children

With the interest in the differences of SNP between the groups in sequence, we furtherly screened BiasSNPs whose variation were dominant in one group/one time point among more than 60% of the individuals. Comparing with the SNPs before and after 30 days of intervention, the detected BiasSNPs in PWS was 4,465, larger than 303 in SO. The PWS and SO had only 81 BiasSNPs in common distributed in 69 genes. Source track indicated that 78 common BiasSNPs were from *F. prausnitzii*, and the remaining three were from *Streptococcus thermophilus*, suggesting that *F. prausnitzii* was the most affected under the intervention.

**FIGURE 3:**
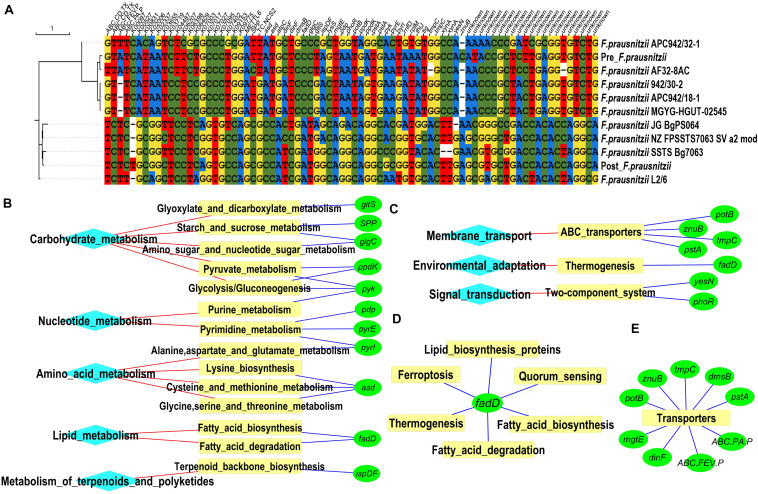
Strains and metabolic pathways corresponding to common BiasSNPs. **(A)** Phylogenetic relationship of nine *Faecalibacterium prausnitzii* and the corresponding strains of pre- and post-intervention groups based on the common BiasSNPs. **(B)** Metabolic pathways related to nutrition metabolism. **(C)** Metabolic pathways associated with environmental adaptation. **(D)** Metabolic pathways associated with the gene *fadD*. **(E)** Genes encoding transporters.

In order to identify the source of these BiasSNPs at genome level, 103 genomes of *F. prausnitzii* were downloaded from the GenBank database, and the nucleotide sites corresponding to common BiasSNPs were abstracted. Only nine out of the 103 *F. prausnitzii* strains had more than 70% coverage of BiasSNPs. Then, we constructed a phylogenetic tree of these nine *F. prausnitzii* and the corresponding strains from the pre- and post-intervention groups based on these 78 BiasSNPs. It was observed that *F. prausnitzii* AF32-8AC was closest to the pre-intervention group, followed by *F. prausnitzii* 942/30-2, *F. prausnitzii* APC942/18-1, and *F. prausnitzii* MGYG-HGUT-02545 in the phylogenetic tree (Figure 3A), while the closest to the post-intervention group was *F. prausnitzii* SSTS Bg7063, followed by *F. prausnitzii* JG BgPS064 and *F. prausnitzii* NZ FPSSTS7063 SV a2 mod. This suggested that the dominant strains of *F. prausnitzii* were converted from *F. prausnitzii* AF32-8AC and *F. prausnitzii* 942/30-2 to *F. prausnitzii* SSTS Bg7063 and *F. prausnitzii* JG BgPS064 after the dietary intervention.

Of the 81 common BiasSNPs, 53 were nonsynonymous mutations in which 51 were in *F. prausnitzii* and the remaining two were in *S. thermophilus*. These 53 nonsynonymous BiasSNPs existed in 49 genes, whose detailed information are listed in Table 1. The enriched KEGG metabolic pathways based on these 49 genes showed that these SNPs were mainly related to nutrition metabolism and environmental adaptation functions (Figures 3B,C). In detail, pathways related to nutrition metabolism included carbohydrate metabolism (ko00720), nucleotide metabolism (ko09104), amino acid metabolism (ko09105), lipid metabolism (ko09103), and metabolism of terpenoids and polyketides (ko09109). Particularly, there were more genes related to carbohydrate and nucleotide metabolism (Figure 3B). Pathways associated with environmental information processing included membrane transport (ko09131), environmental adaptation (ko09159), and signal transduction (ko09132), which were mainly linked with ABC transporters (ko02010), thermogenesis (ko04714), and two-component system (ko02020), respectively (Figure 3C).

**TABLE 1:** Information of 49 genes with nonsynonymous BiasSNP.

GeneID	Name	Definition
SZEY-27A_GL0066464	*ABC.CD.TX*	HlyD family secretion protein
MH0423_GL0087716	*ABC.FEV.P*	Iron complex transport system permease protein
MH0204_GL0062877	*ABC.PA.P*	Polar amino acid transport system permease protein
O2.UC34-2_GL0007607	*ACSL, fadD*	Long-chain acyl-CoA synthetase
MH0094_GL0105570	*asd*	Aspartate-semialdehyde dehydrogenase
SZEY-58A_GL0041727	*ATPF1E, atpC*	F-type H+-transporting ATPase subunit epsilon
NOM017_GL0035853	*bmpA, bmpB, tmpC*	Basic membrane protein A and related proteins
SZEY-106A_GL0033468	*dmsB*	Anaerobic dimethyl sulfoxide reductase subunit B
MH0161_GL0016845	*E3.2.1.8, xynA*	Endo-1,4-beta-xylanase
MH0161_GL0083263	*glgC*	Glucose-1-phosphate adenylyltransferase
T2D-109A_GL0053344	*GLU, gltS*	Glutamate synthase (ferredoxin)
250twins_37179_ GL0047337	*ispDF*	2-C-methyl-D-erythritol 4-phosphate Cytidylyltransferase/2-C-methyl-D-erythritol 2,4-Cyclodiphosphate synthase
BGI-28A_GL0080202	K07027	Glycosyltransferase 2 family protein
MH0260_GL0085944	K09153	Small membrane protein
MH0427_GL0005657	*KARS, lysS*	Lysyl-tRNA synthetase, class II
V1.FI16_GL0163211	*METTL6*	Methyltransferase-like protein 6
MH0089_GL0042766	*mgtE*	Magnesium transporter
NLM015_GL0035022	*pdp*	Pyrimidine-nucleoside phosphorylase
MH0222_GL0152632	*phoR*	Two-component system, OmpR family, phosphate regulon sensor histidine kinase PhoR
BGI-06A_GL0076090	*PK, pyk*	Pyruvate kinase
T2D-59A_GL0116703	*potB*	Spermidine/putrescine transport system permease protein
MH0251_GL0137853	*ppdK*	Pyruvate, orthophosphate dikinase
MH0055_GL0043341	*pstA*	Phosphate transport system permease protein
MH0069_GL0033002	*pyrE*	Orotate phosphoribosyltransferase
T2D-56A_GL0037409	*pyrI*	Aspartate carbamoyltransferase regulatory subunit
250twins_36674_ GL0060378	*rnfD*	Na+-translocating ferredoxin:NAD+ oxidoreductase subunit D
SZEY-103A_GL0004639	*RP-L13, MRPL13, rplM*	Large subunit ribosomal protein L13
V1.CD6-0-PT_GL0047319	*SPP*	Sucrose-6-phosphatase
SZEY-90A_GL0013477	*TC.MATE, SLC47A, norM*	Multidrug resistance protein, MATE family
MH0176_GL0049322	*TC.NCS2*	Nucleobase:cation symporter-2, NCS2 family
MH0422_GL0084041	*thiJ*	Protein deglycase
160400887-stool1_196973	*tig*	Trigger factor
T2D-10A_GL0004234	*yesN*	Two-component system, response regulator YesN
763678604-stool1_204596	*znuB*	Zinc transport system permease protein
MH0136_GL0032411	K07003	Uncharacterized protein
N084A_GL0010742	K07017	Uncharacterized protein
V1.UC35-4_GL0167766	K07095	Uncharacterized protein
T2D-198A_GL0043098	K09775	Uncharacterized protein
V1.FI20_GL0181809	Unclassified	Unclassified
DOM026_GL0058508	Unclassified	Unclassified
MH0136_GL0100087	Unclassified	Unclassified
O2.UC34-2_GL0069427	Unclassified	Unclassified
V1.UC27-0_GL0047860	Unclassified	Unclassified
V1.CD2-0-PN_GL0116497	Unclassified	Unclassified
N051A_GL0048400	Unclassified	Unclassified
264199.stu_r17	Unclassified	Unclassified
MH0094_GL0121652	Unclassified	Unclassified
NLF010_GL0004489	Unclassified	Unclassified
MH0184_GL0028587	Unclassified	Unclassified

Notably, some genes, such as *pyk* and *fadD*, were involved in multiple metabolic pathways (Figures 3B,D), suggesting their important roles in the entire metabolic network. The gene *pyk* encodes pyruvate kinase (Gubler et al., 1994), mainly taking part in glycolysis/gluconeogenesis (ko00010), purine metabolism (ko00230), and pyruvate metabolism (ko00620). The gene *fadD* encodes long-chain acyl-CoA synthetase that can use long-chain fatty acids as carbon source and energy (Pech-Canul et al., 2020), and is mainly involved in fatty acid biosynthesis and degradation (ko00061 and ko00071), ferroptosis (ko04216), lipid biosynthesis proteins (ko01004), quorum sensing (ko02024), thermogenesis (ko04714), etc.

There were several gene-encoding transporters in enrichment pathways (Figure 3E). These transporters include a variety of proteins that are involved in signal transduction and various intracellular processes, such as cell proliferation and differentiation. In this study, the genes with common BiasSNPs encode a variety of transport system permease proteins, such as spermidine/putrescine transport system permease protein (*potB* encoding), phosphate transport system permease protein (*pstA* encoding), polar amino acid transport system permease protein (*ABC.PA.P* encoding), iron complex transport system permease protein (*ABC.FEV.P* encoding), zinc transport system permease protein (*znuB* encoding), etc. Besides, they also encode basic membrane protein A (*bmpA* encoding), multidrug resistance protein (*dinF* encoding), and anaerobic dimethyl sulfoxide reductase subunit B (*dmsB* encoding). These results indicated that these genes with nonsynonymous SNPs were closely related to the transporters under the dietary intervention.

### Prader–Willi Syndrome-Specific BiasSNPs Affected by High-Fiber Dietary Intervention

**FIGURE 4:**
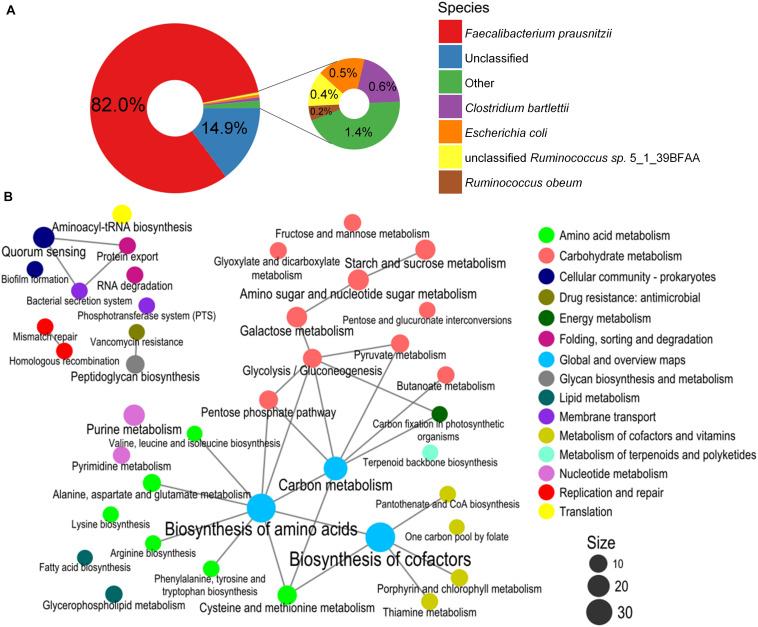
Species composition and enrichment metabolic pathways carrying the BiasSNPs in PWS children. **(A)** Composition of species with BiasSNP in PWS children. Colors represent different species. **(B)** The relationship between the metabolic pathways of BiasSNP in PWS children. The line indicates that the metabolic pathways share the same gene, the dot represents the pathway, the size of the dot represents the number of genes with BiasSNP in the pathway, and the color represents the class of pathway.

PWS-specific BiasSNPs (4,384) were detected between the pre- and post-intervention groups, which were distributed in 2,039 genes and sourced from 34 strains. The distribution at strain level indicated that most of the BiasSNPs were derived from *F. prausnitzii* (82.0%) (Figure 4A).

A network of the KEGG metabolic pathways, with the nonsynonymous BiasSNPs uniquely occurring in the PWS children, was constructed. This network showed that these BiasSNPs were mainly relevant to nutrition metabolism and environmental adaptation (Figure 4B). There were more BiasSNPs in PWS than in SO, and the metabolic functions of these PWS-specific BiasSNPs were similar to that of the common BiasSNPs, indicating that the gut microbial SNPs were more susceptible by dietary intervention in PWS. Nutrition metabolism mainly included carbohydrate metabolism, amino acid metabolism, metabolism of cofactors and vitamins, nucleotide metabolism, and lipid metabolism. Pathways of environmental adaptation mainly covered cellular community, membrane transport, and folding, sorting, and degradation. Genes (242) with nonsynonymous BiasSNPs in the pathway network of PWS children are listed in Supplementary Table 1.

### Simple Obese-Specific BiasSNPs Affected by High-Fiber Dietary Intervention

**FIGURE 5:**
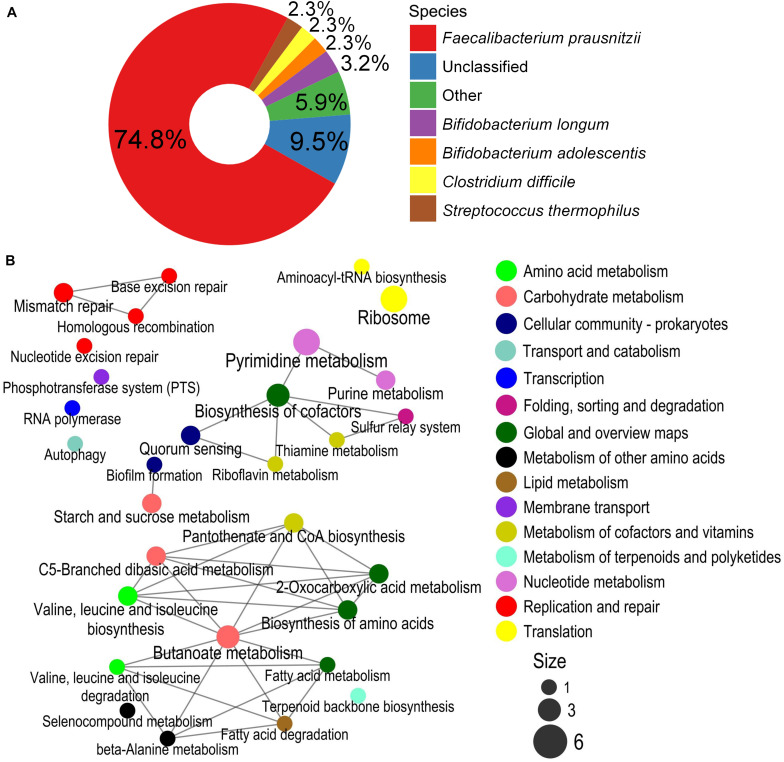
Species composition and enrichment metabolic pathways carrying the BiasSNPs in SO children. **(A)** Composition of species with BiasSNP in PWS children. Colors represent different species. **(B)** The relationship between the metabolic pathways of BiasSNP in PWS children. The line indicates that the metabolic pathways share the same gene, the dot represents the pathway, the size of the dot represents the number of genes with BiasSNP in the pathway, and the color represents the class of pathway.

There were 222 SO-specific BiasSNPs between before and after dietary intervention, far fewer than those in PWS. These BiasSNPs were distributed in 196 genes and sourced from 17 strains, also mainly from *F. prausnitzii* (74.8%) (Figure 5A). Among them, 104 BiasSNPs were nonsynonymous. The network of enriched KEGG metabolic pathways constructed with the genes carrying nonsynonymous BiasSNPs showed that these BiasSNPs were mainly related to nutrition metabolism, DNA replication and repair, and translation (Figure 5B). The nutrition metabolism included carbohydrate metabolism, amino acid metabolism, metabolism of cofactors and vitamins, and nucleotide metabolism. The SNPs related to DNA replication and repair may furtherly influence the genetic variation, including base excision repair, homologous recombination, mismatch repair, and nucleotide excision repair. In addition, a number of nonsynonymous BiasSNP existed in translation-related metabolic pathways, such as aminoacyl-tRNA biosynthesis and ribosome. Twenty-six genes with nonsynonymous BiasSNPs in the pathway network of SO children are presented in Supplementary Table 2.

## Discussion

Our previous study performed a dietary intervention trial on obese children with PWS and SO, and found that the high-fiber diet had improved significantly the physiological indexes of all the obese children and changed the composition and structure of the gut microbiota (Zhang et al., 2015). This study focused on the gut microbial SNP variations that occurred in genes, trying to figure out important genes and functional strains influenced by the intervention.

We found that the remarkable changes in gut microbial SNPs caused by the intervention were related to nutrition metabolism, including carbohydrate metabolism (e.g., gluconeogenesis and pyruvate metabolism), amino acid metabolism, and lipid metabolism in all obese children. This result was not surprising because the SNPs existed densely in strains to adapt to environmental changes. Compared with the normal diet, the WTP diet provides large quantities of whole-grain mix that is rich in starch, soluble and insoluble dietary fiber, protein, and amino acids, but contains a small amount of fat (Xiao et al., 2014). When this excess and/or indigestible nutrition reached the colon, they brought environmental pressures to the microbiota that stayed there. This pressure could facilitate the utilization of indigestible nutrition by causing microbial SNPs and, thus, affecting the functions of the related genes (such as *pyk*, coding the pyruvate kinase). As a result, the metabolic efficiencies of indigestible nutrition substrates would be enhanced to adapt to the shifted environment better. Conversely, as the WTP diet is low in fat, the lower lipid substrate level in the intestinal environment might lead to SNPs in lipid metabolism-relevant genes (such as *fadD*, coding long-chain acyl-CoA synthetase) and, thus, would furtherly reduce the efficiencies of lipid nutrition substrates.

Meanwhile, SNPs also emerged in some pathways related to the adaptability to environmental changes and the virulence of bacteria, such as the two-component system, transport system, secretion system, and drug tolerance. The two-component system is a signal transduction system widely existing in bacteria, which plays an important role in responding to the constantly changing environment by means of protein phosphorylation (Wang et al., 2002; Zuniga et al., 2011). ABC transport system utilizes the energy released by ATP hydrolysis to transport various substrates across membranes, including amino acid, sugar, lipid, polypeptide, alcohol, metal, drug, etc. (Koster, 2001; Hollenstein et al., 2007). Additionally, the ABC transport system is also involved in some other biological processes, such as RNA translation and DNA repair (Licht and Schneider, 2011). HlyD, a member of the membrane fusion proteins (MFP), links the inner and outer membranes in some way by spanning the periplasm and is necessary for the secretion of repeats in toxin (RTX) hemolytic toxins (Pimenta et al., 1999; Pimenta et al., 2005). RTX is a kind of high-molecular weight protein, heat-resistant, calcium-dependent toxin, secreted by a large class of Gram-negative pathogens, which can lysis various creatural target cells (Lally et al., 1999). Dietary changes brought drastic variations to the intestinal environment and intense evolutionary pressure on the gut microbiota in obese children. Though further validation is needed, our results implied that, in response to this environmental pressure, some gut microbial SNPs that occurred might affect the efficiency and function of metabolic pathways related to environmental adaptation, and might be relevant to the health promotion of the obese children.

Previous studies had observed that *Bifidobacterium* was the selectively promoted genera under the WTP intervention due to their outperforming ability to utilize carbohydrates (Xiao et al., 2014; Zhang et al., 2015; Zhao et al., 2018). Indeed, *Bifidobacterium* became dominant strains responsible for nutrition metabolism after the intervention in both PWS and SO children based on SNP density analysis. *Bifidobacterium* has been demonstrated to have various probiotic effects on the health of the host, involving protection of the intestinal barrier, modulation of the immune response, and effects of antimicrobial, anti-inflammatory, and anti-obesity (Marteau et al., 2001; Heuvelin et al., 2009; Turroni et al., 2014). However, when we turned to BiasSNP analysis, unexpectedly, it was *Faecalibacterium*, instead of *Bifidobacterium*, that had the most nonsynonymous SNPs, suggesting that the intervention mainly affected the functional mutations of *Faecalibacterium*, especially *F. prausnitzii*. *F. prausnitzii* can reduce the synthesis of colonic pro-inflammatory cytokines, induce the secretion of anti-inflammatory cytokines, and inhibit the activation of NF-κB and the production of IL-8 (Sokol et al., 2008; Miquel et al., 2015), and produce butyrate to make protective and anti-inflammatory effects (Ohira et al., 2017). In addition, the track to strains carrying BiasSNPs showed that the dominant strains, *F. prausnitzii* AF32-8AC and *F. prausnitzii* 942/30-2, converted to *F. prausnitzii* SSTS Bg7063 and *F. prausnitzii* JG BgPS064 after the dietary intervention, indicating that dietary intervention probably changed the dominant strains of *F. prausnitzii* by changing the intestinal environment. If the study only focuses on the composition or abundance of the gut microbiota as the mainstream used, the important information about *F. prausnitzii* would be neglected. A previous study of 31 *F. prausnitzii* genomes reported that the functional differences among these strains were mainly concentrated in the metabolism of carbohydrates and amino acids (Fitzgerald et al., 2018). However, we found that the functional differences of *F. prausnitzii* were not only on nutrition metabolism but also in response to environmental changes, such as signal transduction and membrane transport. This additional finding suggested that SNP analysis on gut microbiota could provide more details about the functions and characteristics at the strain level.

Some differences in the gut microbial SNP existed between PWS and SO children in response to the intervention. There were more BiasSNPs between before and after intervention in PWS children than in SO, suggesting that the influence of high-fiber diet on the gut microbial SNP may be greater in PWS than in SO. PWS-specific BiasSNPs were mainly related to nutrition metabolism, protein transport, and environmental adaptation. The phosphotransferase system (PTS) in bacteria can transport carbohydrates into cells by phosphorylation (Deutscher et al., 2007), and also perceive available carbohydrates and intracellular energy, regulate the decomposition of metabolites, and ensure the optimal utilization efficiency of carbohydrates in a complex environment (Lengeler, 1996; Kotrba et al., 2001). N-acetylgalactosamine-specific PTS, which correlated with the nonsynonymous BiasSNPs in PWS, is a common amino-sugar transport system in the gut microbiota, which can regulate and transport acetyl galactosamine (Brinkkotter et al., 2000; Ezquerro-Saenz et al., 2006). Additionally, PWS-specific BiasSNPs were also concentrated in two kinds of proteins translocation systems, general secretory (Sec) pathway and twin-arginine translocation (Tat) system. The Sec pathway is a common and universal protein translocation system, which could integrate synthetic proteins into bacterial cell membranes (Zhou et al., 2014; Tsirigotaki et al., 2017), while the Tat system can transport folded proteins efficiently across the cytoplasmic membranes (Palmer et al., 2010). Moreover, PWS-specific BiasSNPs were also related to spore formation, which is wrapped by a layer of complex macromolecular protein shell under special conditions to resist the hydrolysis of enzyme and protect the active molecules (Setlow, 2003; Kim et al., 2006). These differences in gut microbial SNPs between PWS and SO could not be discovered if only composition information was used, which emphasized the importance of SNP analysis again.

In this work, the identified non-synonymous SNPs were dominantly carried by *F. prausnitzii* strains. Though *F. prausnitzii* were well known for their biodiversity, we could not find functional reports about these SNPs. Future efforts are needed to verify/discern the specific effects of these SNPs on the encoded protein activity, their role on metabolism under high-fiber dietary intervention, and their potential beneficial or detrimental influences on host health. The verification/discernment could be done through combining molecular simulation or experimental design.

## Conclusion

Our results demonstrated that the high-fiber dietary intervention altered the gut microbial SNP patterns in obese children, and intervened the efficiency and function of metabolic pathways in nutrition metabolism and environmental adaptation. *F. prausnitzii* had been screened out as the dominant strains by changing multiple functional SNPs under the intervention, which had the potential to improve obesity and could be used as a probiotic supplementary in the prevention and treatment of obesity. This bioinformatics study provided evidence for the influence of dietary intervention on gut microbial SNPs, highlighted the importance of SNP analysis on searching differential genes and functional strains from complexed microbial ecosystem, and gave some enlightenment for obesity or other disease treatment.

## Data Availability Statement

The original contributions presented in the study are included in the article/Supplementary Material, further inquiries can be directed to the corresponding author/s.

## Ethics Statement

The studies involving human participants were reviewed and approved by the Chinese Clinical Trial Registry. Written informed consent to participate in this study was provided by the participants’ legal guardian/next of kin.

## Author Contributions

MZ, LZ, and HL contributed to conception and design of the study. HL performed the statistical analysis and wrote the first draft of the manuscript. All authors contributed to the manuscript revision and approved the submitted version.

## Conflict of Interest

The authors declare that the research was conducted in the absence of any commercial or financial relationships that could be construed as a potential conflict of interest.

## Footnotes

1 https://www.evolgenius.info/evolview
