# Supplementary figures and images for: Gut Microbial SNPs Induced by High-Fiber Diet Dominate Nutrition Metabolism and Environmental Adaption of Faecalibacterium prausnitzii in Obese Children
Source: Front Microbiol. 2021 May 31;12:683714. doi: 10.3389/fmicb.2021.683714 (PMC8200495; doi:10.3389/fmicb.2021.683714)

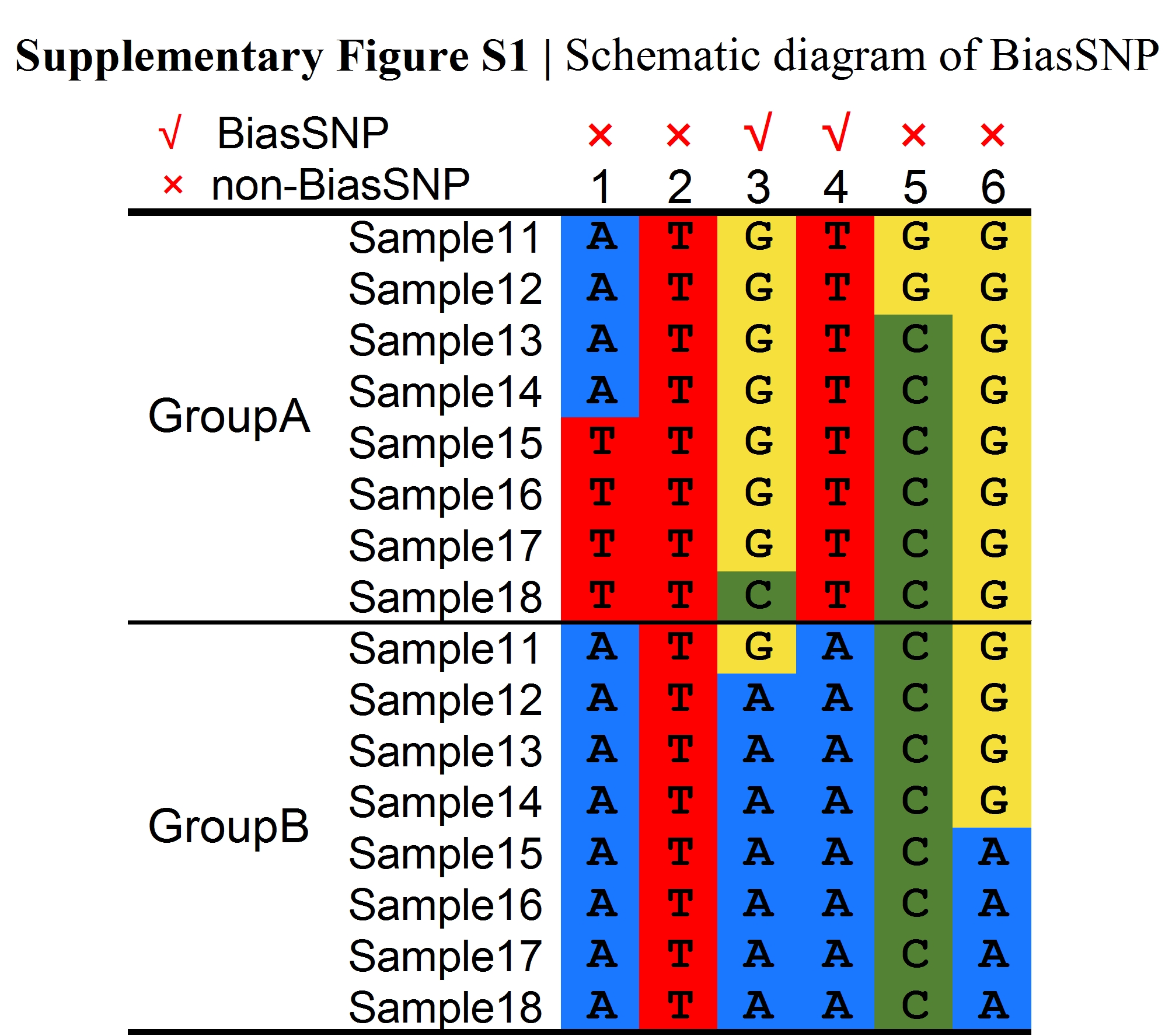

Supplement: Supplementary file 1 [file Image_1.JPEG]
